# Climate risk perception and pharmaceutical supply chain resilience in China

**DOI:** 10.3389/fpubh.2026.1952283

**Published:** 2026-09-11

**Authors:** Shipeng Wang, Han Zhuo, Hui Zhang

**Affiliations:** 1 School of Business, Shandong Normal University, Jinan, Shandong, China; 2 Beijing Technology and Business University, Beijing, China; 3 College of Public Management, Shandong University of Finance and Economics, Jinan, Shandong, China

**Keywords:** climate risk perception, GMP constraints, green innovation, pharmaceutical industry, supplier relationships, supply chain resilience

## Abstract

Climate risk shocks to the global supply chain are becoming more severe, and the pharmaceutical industry is especially vulnerable, given the high stakes of patient safety, cold-chain constraints, geographic concentration of active pharmaceutical ingredients, and stringency of quality regulation. Using panel data on pharmaceutical manufacturers listed in the A-share from 2003 to 2024, we construct a climate risk perception indicator based on the textual analysis of annual reports and examine its impact on the supply chain resilience of firms and the mechanisms involved. Our findings show that the heightened climate risk perception significantly enhances supply chain resilience. This relationship is robust to alternative dependent and independent variables, alternative specifications of fixed effects, and different trimming ranges. Three mechanisms drive the effects: (i) promoting green innovation, (ii) deepening supplier relationships, and (iii) diversifying customer concentration. The heterogeneity analyses suggest that the effects are stronger for state-owned enterprises, smaller firms, and firms with higher managerial risk aversion. This study connects climate risk perception and supply chain resilience in a regulated pharma context, offering new theory and empirical evidence for resilience in relationship-driven supply chains.

## Introduction

Climate change is regarded as one of the most systemic and long-term risks to the global economy in the 21st century (1). Extreme weather events, failure of climate action and natural disasters have always appeared in the list of the top threats by the World Economic Forum in terms of probability and impact (2). Physical climate risks are becoming more frequent and intense, while transition risks are increasing as countries advance on their decarbonization commitments (3–5). For instance, climate-related physical disasters could result in annual fixed asset losses ranging from US$560 billion to US$610 billion for listed firms by 2035 (6). Against this background, the way firms perceive and respond to climate risk matters not only for their own operating stability but also for the resilience and continuity of the supply networks embedded within their ecosystems following external shocks (7). Hence, supply chain resilience, which is the ability of a firm to resist, adapt and recover from disruptive events, has emerged as a crucial dimension to measure climate adaptability (8–10). However, the current literature has focused largely on generic manufacturing contexts, with comparatively little attention to highly regulated industries with distinctive supply architectures, most notably pharmaceuticals. This brings into question the degree and type of climate risk perception and response mechanisms in such regulated settings.

The resilience of pharmaceutical supply chains has different implications than for general manufacturing (11). On the other hand, drug production is under strict Good Manufacturing Practice (GMP) quality management, regulatory audits by agencies the U. S. Food and Drug Administration (FDA) and China National Medical Products Administration (NMPA), and supplier qualification requirements. Switching supplier requires long auditing and validation process (12, 13). On the other hand, China and India are highly concentrated in active pharmaceutical ingredients, cold-chain transport is essential for temperature-sensitive products, and product shelf lives are short, which has direct implications for patient safety; any supply disruption can trigger shortages and public health crises. The Covid-19 pandemic has laid bare many of the fault lines in global pharmaceutical supply chains, and recent extreme weather events have repeatedly disrupted regions that produce APIs, exposing deep systemic fragility (14). For instance, the COVID-19 pandemic revealed the fragility of many of these systems, as over 80% of nations had major interruptions in their drug supply chains (15). These factors suggest that the channels through which climate risk affects the resilience of pharmaceutical supply chains may be very different from those in less regulated industries, calling for dedicated theory and empirical work.

Theoretically, there are two streams of research. Climate risk perception and supply chain resilience have generated a large body of knowledge but are largely siloed. The studies on climate risk perception focus on the effects of climate risks on firms’ financial costs (16), investment behavior (17), green innovation (18), and market valuations (19). Its core variables tend to be around carbon emissions, ESG performance or financial decision making with little extension to supply chain considerations. By comparison, studies on supply chain resilience mostly draw on operations and strategic management theories, with digitalization, partnerships, spare capacity and operational agility widely recognized as key boosters of resilience (20, 21). Most papers simply regard climate risks as external shocks beyond firms’ control and advance planning. Even though a handful of existing work splits climate risks into physical and transition types and compares their economic impacts, few explore how these risks alter supply chain resilience (18).

The listed pharmaceutical manufacturers in A-share market from 2003 to 2024 are selected as the sample of this study and panel fixed effect model is adopted. Empirical results show that firm-level climate risk perception significantly improves the supply chain resilience of pharmaceutical manufacturers, and this finding is robust across various specifications and alternative instrumental variable estimates. Mechanism analysis indicates that climate risk perception enhances resilience through three channels including green innovation, supplier relationship, and customer concentration. Notably, climate risk perception raises supplier concentration, a result that may appear at odds with traditional risk-diversification theories but reflects the pharmaceutical sector’s unique constraints under GMP traceability and supplier qualification regimes: firms preferentially deepen ties with a limited set of audited, qualified suppliers rather than expanding the supplier base amid high uncertainty. The results of the heterogeneity tests demonstrate that the positive effect on supply chain resilience is larger for state-owned enterprises, smaller firms and firms with a higher executive risk appetite. Further risk-type analyses show that it is physical risk perception that mainly drives the improvement of resilience, while transition risk perception has no significant impact. This is in line with the sector’s high dependence on the origin of raw materials, the cold-chain and transport conditions.

Therefore, this paper provides the following three key contributions. First, in theoretical aspect, we integrate climate risk perception with the supply chain resilience literature in the pharmaceutical industry, revealing a distinct adjustment logic of relationship-based supply chains under regulatory constraints. This extends prior work that largely concentrates on general manufacturing (22–24), by highlighting how GMP traceability and supplier qualification regimes shape resilience-focused managerial responses.

Second, in mechanism dimension, this paper identifies and explain asymmetric effects of climate risk perception on supplier concentration and customer concentration, grounded in GMP traceability and supplier qualification constraints. Compared with qualitative analysis or case analysis (25–27), this sector-specific mechanism reconciles empirically observed concentration with risk-diversification theory, enriching the repertoire of supply chain restructuring pathways under climate risk.

Third, from a policy standpoint, this paper puts forward targeted suggestions on climate adaptation and supply chain governance for regulated sectors based on our empirical results. Different from macro policy insights (28–30), our evidence that physical climate risks play a dominant role alongside varied moderating effects can support policymakers in rolling out coordinated rules, so as to strengthen the overall resilience of pharmaceutical supply chains under supervision.

The remaining sections are organized as below. Section 2 is literature review and hypothesis. Section 3 is research design contains data source, variable selection, and model set. Section 4 presents the empirical results. The last section introduces conclusion and offer policies.

## Literature review

### Economic consequences of climate risk perception

Climate risk perception refers to management’s cognition and judgment about the adverse impacts that climate change may impose on operations. Measurement has shifted from indirect proxies to direct textual analysis. Early work relied on carbon intensity, emissions, or ESG scores, which fail to distinguish physical from transition risks or capture managers’ subjective heterogeneity. Sautner et al. (31) combine for the first-time annual earnings conference call texts with topic dictionaries of climate policy, disclosure rules, and IPCC reports. Li et al. (18) further classifies climate risks into acute and chronic physical risks and transition risks. Transition risks are priced in at the level of capital markets; physical risks are more firm-specific.

Three strands dominate the literature on the economic consequences of climate risk perception. First, climate risks and financing/capital structure: high-emission firms face higher bond spreads and loan costs (16), and climate risk amplifies information asymmetry and financing constraints, prompting shifts in asset and liability maturity (24). Second, climate risks and investment/innovation: exposed firms with higher transition risk cut R&D and employment, while proactive responders increase capex and green patenting, highlighting managerial initiative as a key moderator; climate risk also drives greater long-term ESG investments (32). Third, climate risks and market valuation/risk pricing: institutional investors view climate risk as an undiversifiable systematic risk (33), with higher carbon intensity associated with higher expected returns as compensation (19).

However, much of the work has been on internal financial decisions and market performance, with little on how climate risks are shifting external supply chain relationships and resilience. Indeed, climate risk can undermine networks via physical disasters and indirectly change configuration through policy change and changing preferences. Pankratz et al. (34) confirm supply chain transmission channels and demonstrate that extreme temperatures decrease operating revenue, in part due to reduced supplier-output.

### Supply chain resilience

Supply chain resiliency is the ability to resist disruptions, recover quickly and adapt to new conditions when faced with disruptive events (8, 35). Christopher and Peck (9) essentially deconstructed resilience into four dimensions, namely engineering, collaboration, organization and risk management to establish a multi-dimensional measurement basis. Pettit et al. (20) proposed a framework that highlights a transition from resistance to recovery and to adaptability. Measurements typically create composite indices across dimensions of resistance, recovery, and adaptive capacity, using multi-indicator systems and objective weighting methods (entropy, PCA) (21).

Antecedents of resilience are analyzed at three levels: firm capabilities, supply chain structure, and external environment. At the firm capability level, dynamic capabilities explain resilience formation: sensing changes, seizing opportunities, and reconfiguring resources (36). Digital capabilities, as a contemporary form, have been shown to enhance supply chain visibility and response speed (37). At the supply chain structure level, factors such as supplier and customer concentration, supply chain length, and geographic distribution directly influence resilience; reducing concentration is traditionally viewed as beneficial (38), though recent work explores resilience in relational chains under specific contexts (39). At the external environment level, institutional pressures, regulations, and technological change shape firms’ adjustment scopes (40).

### Pharmaceutical supply chain characters

Pharmaceutical supply chains and general manufacturing have five key differences, and these differences define the impact of climate risks on resilience. Good Manufacturing Practice (GMP) constraints First of all the FDA 21 CFR Part 211 and the EU GMP Chapter 5 require documented supplier qualification systems. On-site audits, material validation, stability testing and formal change control with regulatory notifications are required when bringing on a new supplier, which slows down diversification and reconfiguration in response to climate shocks. Second, geographic concentration of API production: Global API capacity is concentrated in China and India, which supply about 70% of APIs. This concentrates risk, with API supply being highly vulnerable to climate events in those hubs and allowing disruption to cascade downstream (41). Third, cold-chain dependence: Many medicines need strict temperature control in transit. Exposure to climate-related disruptions is exacerbated when extreme heat or power outages jeopardize cold-chain integrity (42). Fourth, limited shelf life and continuous demand: short shelf lives do not leave much room for buffering inventory and disruptions quickly translate into shortages and service gaps. Fifth, direct life-safety implications Medicines touch human health, so resilience has huge public health and regulatory implications that go beyond commercial interests.

## Hypothesis development

### Main effect of climate risk perception on supply chain resilience

Based on the theory of dynamic capabilities, firms’ climate risk perception leads to the identification of external environmental changes, discovery of adjustment opportunities and orientation for reconfiguration of supply chain resources. Increased climate risk disclosure by management in annual reports leads to climate considerations being integrated into strategic decision making, making firms more prone to proactively enhance resilience, e.g., by increasing inventory buffers, improving supplier management, and investing in green technologies to reduce the climate sensitivity of production (18, 36). Higher climate risk perceptions also increase the importance of information disclosure and stakeholder communication, reducing information asymmetry among supply chain partners and enhancing coordinated responses (9). Consequently:

*H1*: Controlling for other factors, climate risk perception improves the supply chain resilience of pharmaceutical firms.

### The influencing mechanisms

#### Green innovation capacity

The Porter hypothesis suggests that appropriate environmental regulations and pressures from risk can spur innovation and competitiveness (43). Perceived climate risks lead to energy savings, cleaner production and green material substitution, reducing dependence on climate sensitive resources and improving energy efficiency. Green innovation is at the heart of resilience: proactively addressing transition risks strengthens firms’ green patenting (18). More importantly, supply chain relationships can be stabilized by green technological innovation, which in turn interrupts the negative transmission of climate risk across supply chains (44). Therefore:

*H2a*: Climate risk perception enhances pharmaceutical supply chain resilience by promoting green innovation.

#### Deepened supplier relationships

In pharma, Good Manufacturing Practice (GMP) requirements make supplier qualification more complex, costly, and time-consuming than in general manufacturing. Lengthy audits, validation procedures, quality assessments, and regulatory filings make rapid supplier diversification risky and difficult (12, 13). Consequently, climate risk exposure encourages pharmaceutical firms to deepen cooperation with a core group of pre-qualified suppliers through long-term quality agreements, joint risk-warning systems, and shared inventory and capacity information (45). This relational governance reduces transaction costs and uncertainty, improves coordination, and strengthens resilience to climate-related disruptions, although it may also increase supplier concentration under strict regulatory and institutional constraints. Thus:

*H2b*: Climate risk perception increases pharmaceutical supply chain resilience through deepened Supplier Relationships

#### Customer diversification

Pharmaceutical firms generally face fewer regulatory obstacles to market and customer diversification than on the supplier side. Perceived climate risks may therefore encourage firms to enter new geographic markets, develop new customer segments, and expand or diversify sales channels to reduce dependence on any single customer, market, or region (46). Such diversification enables firms to distribute demand-side risks more broadly and mitigate the impact of localized climate-related disruptions. By lowering customer concentration and reducing exposure to regional demand shocks, diversified demand structures can stabilize revenues, improve market flexibility, and ultimately strengthen the overall resilience of pharmaceutical supply chains. Hence:

*H2c*: Climate risk perception improves supply chain resilience by reducing customer concentration.

### Heterogeneous moderating mechanisms

#### Firm size

The influence of climate risk perception on resilience might depend on firm characteristics. That baseline resilience is higher, big firms are better resourced, more sophisticated in their management and that translates to fewer marginal gains in climate risk perception. Small firms, on the other hand, have fewer options and may be more vulnerable to climate-related changes. Therefore:

*H3a*: Firm size negatively moderates the climate risk–resilience link; smaller firms gain more resilience.

#### Ownership character

In China, ownership structure plays an important role in shaping firms’ responses to climate risks. State-owned enterprises (SOEs) typically benefit from stronger policy support, government coordination, and preferential access to financial and informational resources (47, 48). These advantages can facilitate the translation of climate risk perception into resilience-oriented investments, such as supply chain diversification, inventory buffers, and risk management systems, thereby strengthening their adaptive capacity. Thus:

*H3b*: Ownership type positively moderates the climate risk–resilience link; SOEs benefit more.

#### Executive risk appetite

Executive risk appetite also plays an important role in shaping firms’ strategic responses to climate risks (49). Risk-tolerant leaders are more willing to accept uncertainty and commit resources to forward-looking investments, such as market diversification. By contrast, risk-averse leaders may prioritize short-term stability and avoid costly adjustments. Thus, greater executive risk tolerance can strengthen the translation of climate risk perception into proactive strategies, ultimately enhancing firms’ capacity to absorb disruptions and build long-term resilience. Therefore,

*H3c*: Executive risk appetite positively moderates the climate risk–resilience relationship.

### Differential effects across risk types

Climate risks comprise physical and transition risks with different mechanisms. Physical risks have a direct impact on facilities, supplies and logistics, and have immediate and strong effects on resilience. Transition risks are indirect, longer term impacts driven by changes in policy, technology and preferences (50). In pharma, the effects of physical risks are magnified by exposure to climate-sensitive raw materials, cold-chain integrity and logistics, while the effects of transition risks might be muted by the sector’s relatively low carbon intensity and modest carbon policy exposure. Accordingly:

*H4*: The positive impact of physical risk perception on supply chain resilience is stronger than that of transition risk perception.

## Research design

### Sample and data sources

The paper selects pharmaceutical manufacturing enterprises of A-share listed companies from 2003 to 2024 as research samples, and industry grouping is based on CSRC 2012 classification standard. There are two rationales for the selection of this particular industry. Firstly, the pharmaceutical supply chain is subject to specific restrictive conditions. It is highly regulated by GMP, has geographically concentrated production of active pharmaceutical ingredients, is highly dependent on cold-chain logistics and is closely linked to public health. These features make the industry an important research context for studying the impact of climate risks on supply chain resilience under regulatory constraints. Second, the pharmaceutical sector shows a high exposure to climate hazards. Adverse impacts of climate change amplified by disruptions to raw material production bases and difficulties in maintaining stable temperatures in cold-chain transportation.

The data sources are: basic firm information and financial data are from CSMAR; climate risk perception is measured by the frequency of climate-risk keywords in MD&A sections; green patent data are from the National Intellectual Property Administration; supplier and customer concentration are from the CSMAR Supply Chain Research Database; executive risk appetite is constructed through principal component analysis of financial indicators; historical extreme weather data are from the National Health Commission of China; and the UNDP Human Development Index. We remove firms with ST treatment in any year and winsorize continuous variables at 1st and 99th percentiles to avoid bias and outliers. The final unbalanced panel contains 357 listed pharmaceutical firms, with 3,267 firm-year observations.

### Variables selection

The dependent variable is supply chain resilience (SCR). Following Christopher and Peck (9) and Pettit et al. (20), SCR is measured by a five-dimension index of adaptive capability, resistance capability, recovery capability, human capital, and government support—weighted by entropy to yield a composite SCR. The detailed contents are shown in Table A1. As a robustness check, we use inventory turnover as an alternative proxy for recovery. Specifically, SCR1 is the natural log of (1 + inventory turnover), where operating costs are divided by average net inventory.

The main independent variable is climate risk perception (CRP), which is measured by the frequency of climate-risk keywords in annual MD&A texts, as in Sautner et al. (31) and Li et al. (18). CRP is decomposed into physical climate risk perception (CRPPHY) and transition climate risk perception (CRPTRS) with a dictionary of 830 terms. CRP is ln (1 + frequency). As a robustness check, we use PCRP as the ratio of climate change words to total words in MD&A.

Leverage, profitability, growth, tenure, governance and ownership are the control variables LEV, ROA, INCOME, AGE, Director, Independent, DUAL and INST, respectively. Green innovation (INNO) = ln(1 + green patent applications) and supply chain concentration (SUPC, CUPC) are mediators. SUPC and CUPC measure the extent of purchases from the largest five suppliers and extent of sales to the largest five customers.

The moderators are SIZE (log assets), PROP (1 for SOEs), and risk appetite of management (MRP). Referring to MacCrimmon and Wehrung (51), the MRP is constructed by Principal Component Analysis (PCA) method from five dimensions. (1) Asset structure: risky assets-to-total assets; (2) Solvency: debt-to-asset ratio; (3) Profit structure: core profit ratio; (4) Profit distribution: retention ratio; (5) Cash flow: self-financing ratio and capital expenditure ratio. The full definitions are given in Table 1.

**Table 1:** Variable definition.

Variable category	Variable name	Abbreviation	Measurement
Dependent variable	Supply chain resilience	SCR	Comprehensive measurement via entropy weight method
	Supply chain resilience (robustness)	SCR1	Operating costs/average net inventory balance, taken as logarithm
Independent variable	Climate risk perception	CRP	Climate-related risk word frequency in annual reports + 1, taken as logarithm
	Climate risk perception (robustness)	PCRP	Proportion of climate-related risk word frequency in annual reports
	Physical climate risk perception	CRPPHY	Physical risk word frequency + 1, taken as logarithm
	Transition climate risk perception	CRPTRS	Transition risk word frequency + 1, taken as logarithm
Control variable	Leverage ratio	LEV	Total liabilities/total assets
	Return on total assets	ROA	Net profit/total assets
	Firm growth	INCOME	Revenue growth rate
	Firm age	AGE	Ln(firm age + 1)
	Board size	Director	Ln(number of board directors + 1)
	Proportion of independent directors	Independent	Number of independent directors/total number of board directors
	CEO duality	DUAL	Takes the value of 1 if the CEO also serves as the board chair, and 0 otherwise
	Institutional shareholding ratio	INST	Institutional investors’ shareholdings/total share capital
Mechanism variable	Green innovation	INNO	Number of green patent applications + 1, taken as logarithm
	Supplier concentration	SUPC	Proportion of purchases from the top five suppliers
	Customer concentration	CUPC	Proportion of sales to the top five customers
Moderating variable	Firm size	SIZE	Natural logarithm of total assets
	Ownership nature	PROP	Takes the value of 1 for state-owned enterprises, and 0 otherwise
	Managerial risk preference	MRP	Comprehensive indicator derived from principal component analysis

### Model construction

To test Hypothesis H1, the following two-way fixed-effects panel regression model is established:

$$SCR_{it}=\alpha +\beta CRP_{it}+\gamma X_{it}+\mu_{i}+\lambda_{t}+\epsilon_{it}$$

where $SCR_{it}$ denotes supply chain resilience of firm i in year t; $CRP_{it}$ represents climate risk perception; $X_{it}$ is a vector of control variables; $\mu_{i}$ stands for firm fixed effects; $\lambda_{t}$ denotes year fixed effects; and $\epsilon_{it}$ is the random error term. The core coefficient of interest is β , which captures the impact of climate risk perception on supply chain resilience. A significantly positive β supports Hypothesis H1. Two-way fixed effects simultaneously absorb time-invariant firm heterogeneity and common time-varying shocks, mitigating endogeneity originating from omitted variables to a certain extent.

For mechanism analysis, this paper follows methodological suggestions for mediation testing (52, 53). The identification is based on regressions of mediators on the independent variable. To avoid the estimation bias caused by endogeneity of mediating variables, this study focuses on the impacts of CRP on INNO, SUPC, and CUPC, instead of including mediators into the equation for dependent variable. Heterogeneity and moderation analyses include interaction terms to examine the moderating roles of the firm size, ownership nature and executive risk appetite. We address endogeneity concerns using an instrumental variable approach with two stage least square estimation. All regressions use firm-level cluster-robust standard errors.

## Empirical results

### Descriptive analysis

Table 2 provides descriptive statistics for the main variables. SCR (supply chain resilience) has a mean 2.273 and a standard deviation 1.257, with a minimum of 0.119 and a maximum of 4.599, suggesting considerable cross-firm heterogeneity and sufficient variation for identification. CRP (climate risk perception) has an average value of 3.090 with a standard deviation of 1.910, a minimum of 0, and a maximum of 6.41, indicating broad divergence in attention to climate risks. SCR skewness is −0.545. CRP skewness is −0.258. This indicates a left skewed distribution for SCR and a relatively symmetric distribution for CRP. For example, LEV, ROA and AGE are 0.333, 0.052 and 2.909 in log form for controls, respectively, indicating a prevalence of matured firms. DUAL is 0.319 and INST is 0.456, showing roughly 32% dual-role leadership and significant institutional ownership. The distributions generally are in compliance with realities in the industry and there is no apparent anomaly. This validates the regression analyses to come.

**Table 2:** Variable description.

Variable	*N*	Mean	SD	Min	P25	P50	P75	Max	Skew.	Kurt.
SCR	3,267	2.273	1.257	0.119	1.640	2.571	3.171	4.599	−0.545	2.257
CRP	3,267	3.090	1.910	0.000	1.609	3.296	4.727	6.410	−0.258	1.862
LEV	3,267	0.333	0.186	0.042	0.183	0.311	0.459	0.827	0.543	2.648
ROA	3,267	0.052	0.074	−0.236	0.019	0.052	0.090	0.248	−0.774	6.106
INCOME	3,267	0.154	0.396	−0.594	−0.016	0.110	0.228	2.752	3.607	23.020
AGE	3,267	2.909	0.370	1.792	2.708	2.944	3.178	3.565	−0.764	3.404
Director	3,267	2.255	0.173	1.792	2.079	2.303	2.303	2.773	0.022	4.124
Independent	3,267	0.369	0.048	0.286	0.333	0.333	0.429	0.500	0.901	2.786
DUAL	3,267	0.319	0.466	0.000	0.000	0.000	1.000	1.000	0.775	1.601
INST	3,267	0.456	0.230	0.011	0.280	0.476	0.633	0.904	−0.185	2.126

### Main regression results

Table 3 presents the results of the baseline regression of the impact of climate risk perception (CRP) on supply chain resilience (SCR). Columns (1)–(4) report estimates without fixed effects, with firm fixed effects only, with year fixed effects only and with both firm and year fixed effects. The positive and significant coefficients of the CRP at the 1% level in Columns (1), (3), and (4) indicate that a higher climate risk perception leads to a higher SCR and supports Hypothesis H1. In the two-way fixed effects specification (Column 4), one unit increase in CRP increases SCR by 0.036 units. The magnitude is robust to both time-invariant firm heterogeneity and time-varying shocks. Our finding of a positive relationship contrasts with previous evidence, such as Guo and Li (22), showing that escalating physical climate risks can undermine supply chain resilience. This divergence may arise from the fundamental distinction between climate risk perception and realized physical climate risk. Whereas physical climate risks capture actual climate-related disruptions and exposures, climate risk perception reflects firms’ awareness and assessment of potential climate threats. Heightened risk perception can therefore induce firms to anticipate potential disruptions and undertake proactive measures, such as identifying supply chain vulnerabilities, strengthening contingency planning, diversifying suppliers, and enhancing risk-mitigation capabilities. By enabling firms to prepare for climate-related disruptions before they materialize, climate risk perception may ultimately strengthen supply chain resilience. We also notice that the negative coefficient in Column (2) reflects omitted time varying macro factors. Inclusion of year fixed effects removes these confounding trends and exposes the true effect of CRP. The change is not a sign of instability but confirms the need for controls for the year. Furthermore, after controlling for year effects in Columns (3) and (4), CRP coefficient become significantly positive, which underscores the importance of time-varying controls for causal identification with text-based indicators.

**Table 3:** Basic regression.

Variable	(1)	(2)	(3)	(4)
	SCR	SCR	SCR	SCR
CRP	0.057***	−0.055***	0.035***	0.036***
	(0.013)	(0.018)	(0.012)	(0.013)
LEV	−0.844***	−0.749***	−0.441***	−0.509***
	(0.124)	(0.186)	(0.092)	(0.117)
ROA	3.188***	4.379***	1.568***	1.900***
	(0.319)	(0.377)	(0.226)	(0.241)
INCOME	0.235***	0.122**	0.073*	0.002
	(0.055)	(0.054)	(0.038)	(0.034)
AGE	0.418***	1.505***	−0.236***	0.106
	(0.068)	(0.103)	(0.057)	(0.187)
Director	−0.399***	−0.474**	−0.258***	−0.368***
	(0.142)	(0.216)	(0.099)	(0.135)
Independent	−0.401	0.316	−0.992***	−0.435
	(0.495)	(0.631)	(0.342)	(0.397)
DUAL	−0.126***	−0.000	−0.190***	−0.116***
	(0.046)	(0.057)	(0.032)	(0.036)
INST	−0.462***	0.106	−0.027	0.220
	(0.094)	(0.215)	(0.067)	(0.136)
Constant	2.258***	−1.026	3.927***	2.855***
	(0.473)	(0.710)	(0.334)	(0.693)
*N*	3,267	3,257	3,267	3,257
Year FE	No	No	Yes	Yes
Firm FE	No	Yes	No	Yes
adj. *R*^2^	0.117	0.371	0.581	0.756

Clustered robust standard errors are reported in parentheses. *p < 0.1, **p < 0.05, ***p < 0.01.

### Robustness test

This study uses four strategies for robustness checks to test the robustness of the baseline regression results, and the results are shown in Table 4.

(1) We change the dependent variable. Replacing the dependent variable with the natural log of the inventory turnover ratio (SCR1), reflecting resilience from an operational efficiency perspective. Column (1) shows that CRP is still significantly positively related to SCR1 at the 1% level, indicating that the facilitating effect of climate risk perception on supply chain resilience does not depend on how resilience is measured.
(2) We substitute the core independent variable. To reduce measurement bias from differences in length of reports, PCRP is used to replace the core independent variable, which is the proportion of climate risk keywords to total words in MD&A sections. Column (2) confirms a significantly positive CRP coefficient at the 1% level, thus reinforcing the robustness of the main result.
(3) We include province-year interactive fixed effects. Add province-year interactive fixed effects in addition to the two-way fixed effects. It has region-specific, time-varying enforcement and infrastructure factors. In column (3), the CRP remains significantly positive at the 5% level. Notably, the coefficient of CPR does not change after adding province × year FE. The potential reason is that the CRP mainly stems from firm-specific operational characteristics and industry-level competition, rather than from region-specific macroeconomic fluctuations, such as localized policy shocks. Thus, absorbing the province × year time-varying unobservables does not erode its explanatory power.
(4) We modify the winsorization range. The 2.5th–97.5th percentiles are used as the winsorization range. Column (4) presents a highly positive CRP coefficient at the 1% level, consistent with the baseline results, and reduces the influence of outliers.

**Table 4:** Robustness test.

Variable	(1)	(2)	(3)	(4)
	SCR1	SCR	SCR	SCR
CRP	0.015***		0.036**	0.037***
	(0.005)		(0.016)	(0.013)
PCRP		0.346***		
		(0.081)		
LEV	0.175***	−0.494***	−0.395***	−0.493***
	(0.041)	(0.117)	(0.137)	(0.118)
ROA	0.625***	1.865***	1.632***	2.112***
	(0.084)	(0.240)	(0.271)	(0.269)
INCOME	0.162***	0.001	−0.020	0.007
	(0.012)	(0.034)	(0.039)	(0.049)
AGE	−0.187***	0.144	−0.105	0.059
	(0.065)	(0.186)	(0.233)	(0.195)
Director	0.008	−0.372***	−0.352**	−0.402***
	(0.047)	(0.135)	(0.171)	(0.143)
Independent	0.159	−0.515	−0.519	−0.387
	(0.138)	(0.397)	(0.486)	(0.408)
DUAL	0.002	−0.114***	−0.132***	−0.118***
	(0.012)	(0.036)	(0.042)	(0.036)
INST	0.133***	0.187	0.304*	0.199
	(0.048)	(0.136)	(0.167)	(0.136)
Constant	1.522***	2.845***	3.436***	3.036***
	(0.242)	(0.692)	(0.900)	(0.733)
*N*	3,267	3,257	3,267	3,257
Year FE	Yes	Yes	Yes	Yes
Firm FE	Yes	Yes	Yes	Yes
adj. *R*^2^	0.797	0.757	0.747	0.757

Column (1) replaces the dependent variable with SCR1; column (2) replaces the explanatory variable with PCRP; column (3) adds province × year interaction fixed effects; column (4) extends trimming to 2.5%/97.5%. Cluster-robust standard errors are in parentheses. *p < 0.1, **p < 0.05, ***p < 0.01.

Overall, these four robustness tests corroborate that climate risk perception enhances supply chain resilience among pharmaceutical firms.

### Endogeneity issue: instrumental variables method

Although the baseline regressions and robustness checks control for a large set of firm characteristics and two-way fixed effects, they are still subject to endogeneity problems, including omitted variables, reverse causality, and measurement error. We interact this variable with the average climate risk perception of other pharmaceutical companies in the same area for the same year to generate a Bartik-style interactive instrumental variable with sufficient variation to satisfy the identification requirement for panel data (54), since unprocessed historical extreme weather days are constant over time. In the construction of the instrumental variable, we drop the data of cities with less than three registered pharmaceutical companies to ensure the reliable construction of indicators.

The results of the instrumental variable (IV) estimation are shown in Table 5. The dependent variables are CRP and PCRP in columns (1) and (3) of the first-stage regressions. The coefficients of IV1 and IV2 are positive and statistically significant at the 1% level, indicating that the instruments based on interaction are strongly correlated with firms’ perception of climate risk. The estimates of the second stage are shown in columns (2) and (4). The estimates of both CRP and PCRP remain significantly positive at 5% level. In particular, the estimated coefficient on CRP (0.250) is much larger than the baseline estimate (0.036), consistent with the LATE interpretation. Further, the Cragg–Donald F-statistics of 37.45 and 1637.53 are well above the 10 threshold, suggesting that worries about weak instruments are not likely. The results of the IV analysis confirm the robustness of the main results and alleviate concerns about endogeneity, indicating that higher awareness of climate risk improves SCR for pharmaceutical companies.

**Table 5:** IV regression.

Variable	(1)	(2)	(3)	(4)
	CRP	SCR	PCRP	SCR
IV1	0.051***			
	(0.009)			
CRP		0.250**		
		(0.124)		
IV2			0.254***	
			(0.006)	
PCRP				0.278**
				(0.131)
LEV	0.165	−0.580***	−0.021	−0.524***
	(0.165)	(0.121)	(0.021)	(0.113)
ROA	−0.642*	1.957***	0.008	1.785***
	(0.340)	(0.257)	(0.044)	(0.232)
INCOME	0.040	0.002	0.002	0.012
	(0.048)	(0.035)	(0.006)	(0.033)
AGE	0.713***	0.018	−0.054	0.208
	(0.263)	(0.208)	(0.034)	(0.179)
Director	0.406**	−0.443***	0.041*	−0.352***
	(0.190)	(0.146)	(0.024)	(0.130)
Independent	0.170	−0.421	0.194***	−0.448
	(0.559)	(0.401)	(0.072)	(0.383)
DUAL	−0.109**	−0.081**	−0.021***	−0.104***
	(0.051)	(0.039)	(0.007)	(0.035)
INST	0.148	0.109	0.095***	0.122
	(0.192)	(0.139)	(0.025)	(0.132)
*N*	3,236	3,236	3,236	3,236
Year FE	Yes	Yes	Yes	Yes
Firm FE	Yes	Yes	Yes	Yes
CDWald F	37.450	37.450	1637.532	1637.532

Columns (1) and (3) are first-stage regressions, while columns (2) and (4) are second-stage regressions. IV1 is the mean number of historical extreme weather days × mean CRP of other drug companies in the same area; IV2 corresponds to PCRP. Standard errors are in parentheses. *p < 0.1, **p < 0.05, ***p < 0.01.

### Mechanism test

This research aims at investigating the pathways by which climate risk perception affects supply chain resilience, through the methodological protocols of mechanism evaluation, with a focus on the estimation of the effects of climate risk perception on intermediary variables. The pertinent results are shown in Table 6.

**Table 6:** Mechanism test.

Variable	(1)	(2)	(3)
	INNO	SUPC	CUPC
CRP	0.018**	0.535**	−0.455**
	(0.009)	(0.262)	(0.215)
LEV	0.144*	−2.419	−3.131*
	(0.077)	(2.316)	(1.899)
ROA	0.207	10.301**	9.878***
	(0.158)	(4.469)	(3.666)
INCOME	−0.009	0.671	0.672
	(0.022)	(0.636)	(0.522)
AGE	−0.317***	−8.581**	−15.662***
	(0.122)	(3.786)	(3.106)
Director	−0.037	4.649*	−0.025
	(0.089)	(2.681)	(2.199)
Independent	−0.435*	11.251	−4.733
	(0.260)	(8.136)	(6.674)
DUAL	0.027	0.438	0.855
	(0.024)	(0.682)	(0.559)
INST	0.107	−5.134*	1.618
	(0.089)	(2.731)	(2.240)
Constant	1.245***	44.121***	74.064***
	(0.454)	(14.361)	(11.781)
*N*	3,241	2,673	2,673
Year FE	Yes	Yes	Yes
Firm FE	Yes	Yes	Yes
adj. *R*^2^	0.416	0.660	0.742

Clustered robust standard errors are reported in parentheses. *p < 0.1, **p < 0.05, ***p < 0.01.

#### Green innovation capacity

In this paper, we explore whether climate risk perception (CRP) influences green innovation (INNO) in pharmaceutical companies. CRP significantly enhances INNO at the 5% level in column (1), which supports H2a. This is in line with the Porter hypothesis and Huang et al. (44) research. Environmental challenges lead to research and development for energy efficiency, sustainable production and substitution of eco-friendly materials, thus increasing production flexibility and supply chain resilience. In practice, green innovation matters for APIs, which consume energy and are harmful to the environment. Better green processes reduce reliance on climate-sensitive inputs, and sustainable materials diversify and strengthen supply chains against risks of geographic concentration.

#### Deepened supplier relationships

We examine supplier concentration (SUPC) as a potential mechanism. In column (2) we see that CRP is positively correlated with SUPC at the 5% significance level, consistent with H2b. This result is an example of the characteristics of the pharmaceutical sector. Under GMP rules, it is expensive and time-consuming to switch suppliers, so companies with rising climate risk are more likely to deepen long-term partnerships with qualified suppliers (45). Stable alliances provide resilience in the supply chain through better coordination rather than diversification of suppliers.

#### Customer diversification

We consider customer concentration (CUPC) as a means to do so. H2c: Customer concentration is significantly lower at 5% significance level with CRP. On the customer side, relaxed regulations increase demand for companies by expanding markets, segments and channels to reduce downstream risks (46). Centralized buying gives negotiating power downstream and the perception of climate risk reinforces diversification as a safety net.

### Heterogeneity and moderation effect analysis

Table 7 presents the results of heterogeneity and moderation analyses, which are implemented by introducing interaction terms.

**Table 7:** Heterogeneity and moderation effect.

Variable	(1)	(2)	(3)
	SCR	SCR	SCR
SIZE × CRP	−0.014*		
	(0.008)		
SIZE	0.133***		
	(0.041)		
PROP × CRP		0.042**	
		(0.016)	
PROP		−0.099	
		(0.077)	
MRP × CRP			0.031**
			(0.014)
MRP			−0.082
			(0.055)
CRP	0.335**	0.026*	0.048***
	(0.170)	(0.014)	(0.014)
LEV	−0.574***	−0.491***	−0.438***
	(0.118)	(0.115)	(0.142)
ROA	1.853***	1.825***	2.301***
	(0.241)	(0.236)	(0.263)
INCOME	−0.003	0.005	−0.020
	(0.034)	(0.033)	(0.040)
AGE	−0.013	0.213	0.067
	(0.190)	(0.184)	(0.250)
Director	−0.352***	−0.318**	−0.085
	(0.136)	(0.132)	(0.165)
Independent	−0.400	−0.464	−0.295
	(0.396)	(0.389)	(0.440)
DUAL	−0.114***	−0.106***	−0.111***
	(0.036)	(0.035)	(0.039)
INST	0.176	0.262**	0.166
	(0.138)	(0.133)	(0.157)
Constant	0.311	2.451***	2.405***
	(1.055)	(0.683)	(0.912)
N	3,256	3,250	2,805
Year FE	YES	YES	YES
Firm FE	YES	YES	YES
adj. R2	0.758	0.766	0.702

Clustered robust standard errors are reported in parentheses. *p < 0.1, **p < 0.05, ***p < 0.01.

#### Firm size

Column (1) demonstrates a substantial negative SIZE × CRP interaction at the 10% level, which is consistent with H3a. This suggests a stronger effect of climate risk perception on SCR for smaller enterprises. Large pharma companies have already advanced SCM, plenty of resources and stronger baseline resilience, therefore the room for improvement is less (55). Smaller enterprises, which are more constrained and less resilient initially, get greater marginal benefits from climate-risk-driven adaptations. In practice, laws and oversight should focus on small and medium-sized pharmaceutical companies to maximize the benefits of resilience.

#### Ownership character

Column (2) shows a positive PROP × CRP interaction at the 5% level, supporting H3b. Perception of climate risk is more effective to improve SCR for state owned businesses (SOEs). In China’s institutional environment, SOEs have more social duties, policy guidance and often superior financing, enabling more impactful resilience measures (e.g., supplier checks, green improvements, buffers) (56). This suggests SOEs as pharma climate adaptation leaders. Policy implications include targeted support for non-SOEs and appropriate policy adjustments to reduce the resilience gap across ownership types.

#### Executive risk appetite

Column (3) presents a significant positive interaction of MRP and CRP at 5% significance level (H3c). The association between climate risk awareness and SCR is stronger when high executive risk tolerance is present as anticipatory investments and structural changes are intensified (environmental expenditures, supplier restructuring, wider customer demographics). Expenditures and ambiguity may make cautious managers delay decisions and thereby inhibit communication. The findings underline the importance of governance and incentives that stimulate the right kind of risk-taking to translate climate awareness into tangible resilience investments for pharmaceutical companies (49).

### Climate risk types heterogeneous effect

Table 8 disintegrates the perception of climate risk into transition (CRPTRS) and physical (CRPPHY). Columns (1)–(2) indicate that CRPPHY improves supply chain resilience (SCR) at 1% significance level, whereas CRPTRS is statistically insignificant. Therefore, it supports Hypothesis H4. Results indicate that physical risks have more immediate and direct impacts on API production, cold chains and manufacturing facilities (22), whereas transition risks mainly affect resilience through longer-term policy and technology changes. The pharmaceutical sector is relatively less carbon intensive and direct regulatory pressure related to low-carbon economy is limited, which also explains the moderate impact of transition risk on SCR. In practice, policy should focus on physical-risk mitigation by diversifying API production regions, building cold-chain infrastructure and ensuring emergency drug supply mechanisms are resilient to extreme weather. These results are a validation of the idea of differentiating types of risk in research and policy design to address effectively sector-specific vulnerabilities and resilience strategies, from a theoretical and practical point of view.

**Table 8:** Different effects of climate risk types.

Variable	(1)	(2)	(3)	(4)
	SCR	SCR	SCR	SCR
CRPPHY	0.045***			
	(0.015)			
CRPTRS		0.001		
		(0.026)		
PCRPPHY			0.692***	
			(0.163)	
PCRPTRS				0.179
				(0.247)
LEV	−0.510***	−0.502***	−0.494***	−0.506***
	(0.117)	(0.118)	(0.117)	(0.117)
ROA	1.898***	1.875***	1.865***	1.873***
	(0.241)	(0.241)	(0.240)	(0.241)
INCOME	0.002	0.003	0.001	0.003
	(0.034)	(0.034)	(0.034)	(0.034)
AGE	0.111	0.132	0.144	0.137
	(0.187)	(0.187)	(0.186)	(0.187)
Director	−0.369***	−0.352***	−0.372***	−0.351***
	(0.135)	(0.135)	(0.135)	(0.135)
Independent	−0.441	−0.431	−0.515	−0.439
	(0.397)	(0.397)	(0.397)	(0.397)
DUAL	−0.115***	−0.121***	−0.114***	−0.119***
	(0.036)	(0.036)	(0.036)	(0.036)
INST	0.215	0.225*	0.187	0.219
	(0.136)	(0.136)	(0.136)	(0.137)
Constant	2.845***	2.847***	2.845***	2.814***
	(0.693)	(0.702)	(0.692)	(0.696)
*N*	3,257	3,257	3,257	3,257
Year FE	Yes	Yes	Yes	Yes
Firm FE	Yes	Yes	Yes	Yes
adj. *R*^2^	0.756	0.755	0.757	0.755

Columns (1) and (2) use the log of word frequency, while columns (3) and (4) use the proportion of word frequency. The numbers in parentheses are cluster-robust standard errors. *p < 0.1, **p < 0.05, ***p < 0.01.

## Conclusion and policy implications

This study examines how pharmaceutical manufacturers respond to climate-related risks using a sample of A-share listed firms in China from 2003 to 2024. We develop a climate risk perception measure based on the MD&A sections of annual reports and investigate its effect on supply chain resilience (SCR). The results show that greater climate risk perception significantly improves SCR, with the findings remaining robust across alternative specifications, winsorization, and an instrumental-variable approach using historical extreme weather as a Bartik-style instrument. Further analysis identifies three main channels: greater green innovation, stronger supplier relationships, and lower customer concentration. The increase in supplier concentration suggests that firms may strengthen relationships with qualified suppliers when regulatory requirements, such as GMP traceability, limit rapid supplier switching. The positive effect is larger for state owned enterprises, smaller firms, and firms with more risk-tolerant executives. Importantly, physical rather than transition risk is behind these resilience gains, emphasizing the significance of climate-sensitive raw materials, cold-chain logistics and transport infrastructure. Overall, the results offer more general insights into how firms can adapt their supply chains to the increasing uncertainty of climate.

The present study proposes four policy recommendations. Given the prevalence of physical risks in pharma, adaptation should focus on monitoring and mitigation first. Governments should develop risk maps and extreme weather alerts for API origins, encourage sourcing APIs from different geographical locations and improve cold chain facilities thru the installation of temperature monitoring, back-up power and emergency transport plans. We should also establish pharmaceutical emergency reserves and minimum safety stocks for critical medicines to ensure patient access during climate shocks.

Second, supply-chain adjustment is shaped by GMP requirements. The regulators could set up emergency supplier-qualification procedures, such as pre-approved backup suppliers and fast-track reviews of GMP-certified alternatives, while maintaining quality standards. A centralized supplier database could also help cut down on documentation and switching delays during climate emergencies.

Third, policy support should be heterogeneous across firms. Targeted subsidies for upgrading green equipment, digital supply-chain systems and climate-risk assessments can lower adaptation costs for smaller firms. SOEs can pilot climate-resilient procurement and logistics practices and regulators can disseminate such practices to encourage private firms to strengthen resilience.

Fourth, companies need to embed climate risk into executive incentives and governance. Long-term compensation aligned to green R&D, contingency planning and supplier resilience may reduce short-termism and promote sustained adaptation given the role of executive risk appetite. Regulators may also enhance accountability by encouraging climate-related performance indicators in evaluations of executives.

Limitations include reliance on MD&A-based climate risk perception that may reflect disclosure choices or managerial tone rather than true exposure. In future work we could do cross-validation with earnings calls, CSR reports, and textual data sources. Second, SCR is captured by a composite entropy-weighted index of financial indicators, which might exclude operational aspects; incorporating real-time disruption data and performance measures would enhance validity. Third, this study is focused on the pharmaceutical sector; testing validity in other regulated sectors would test generalizability, and refine policy guidance.

## Data Availability

The raw data supporting the conclusions of this article will be made available by the authors, without undue reservation.

## Appendix

**Table A1:** Evaluation index system for SCR.

Primary indicator	Secondary indicator	Indicator definition
Adaptive capability	Sales level	Operating revenue
	Fund management efficiency	Operating revenue/accounts receivable
	Production capacity	Total number of active employees
	Digitalization level	Degree of digital transformation
Resistance capability	Innovation output	Total number of patents independently obtained in the current year
	Equity multiplier	Total assets/owners’ equity
	Equity-to-debt ratio	Total liabilities/owners’ equity
	Fixed assets	Fixed assets
	Risk-taking level	Three-year rolling standard deviation of industry-adjusted return on total assets
	Supply chain concentration	Average of the sum of procurement and sales proportions to the top five suppliers and customers
Recovery capability	Net profit margin on sales	Net profit/operating revenue
	Return on equity	Net profit/shareholders’ equity
	Inventory turnover	Cost of goods sold/inventory
	Current ratio	Current assets/current liabilities
	Cash generation capacity	The firm’s ability to generate cash flow
	Supplier procurement amount	Total procurement amount from suppliers in the current period
	Customer sales amount	Total sales amount to customers in the current period
Human capital	Bachelor’s degree or above employee ratio	Proportion of personnel with bachelor’s degree or above to total employees
	R&D personnel ratio	Proportion of R&D personnel to total employees
Government support	Government subsidies	Total amount of various government subsidies received
	Income tax payable	Amount of income tax expense
